# Notice of the American Society of Dental Surgeons

**Published:** 1840

**Authors:** 


### THE AMERICAN SOCIETY OF DENTAL SURGEONS.

The extracts from the proceedings of the "*American Society of Dental Surgeons*," contained in this number of the Journal, will, we doubt not, be received by our professional readers with pleasure. The formation of this Society forms a new epoch in the history of this department of Surgery; — it betokens, on the part of those concerned in the getting of it up, a determination to rescue the Science from the neglected condition in which it has so long remained, and to elevate it to a level with the other branches of medicine. That they will succeed in this, we hesitate not to affirm, provided the Society be properly conducted. Having set out with the determination to admit none to membership in it, except such as are respectable and known to be competent to practice the profession, it only remains to carry out this resolution to ensure for the association the beneficial results which its warmest friends have dared to anticipate. For our own part, we are sanguine in the belief that it will be productive of great good both to the profession and community at large. We rejoice, therefore, at its formation, and most fervently do we hope that the objects contemplated by it will be realized.

A National Society of Dental Surgeons being now organized, and we believe, under auspices that promise the most favourable and happy results, we trust that the educated and better informed members of the profession will come forward and unite their efforts with those who have engaged in this most noble and praiseworthy enterprise ; for it is an enterprize which has for its object the advancement of the Science, the dignity and respectability of the avocation, and the good of community. Let not selfishness, jealousy, or sordid ambition, keep any one, having just claims to a scientific and thorough knowledge of the art, away. Nor let any one imagine that by his own individual efforts, however high, as a practitioner, in public estimation, he may stand, can remove from the profession, of which he is a member, the odium that empyricism has fixed upon it. This attaches, more or less, to all, and will continue to do so, until the educated and scientific of their members, shall, as a body put forth their efforts to elevate the standard of professional qualification.—To this end, a Society of this kind seems eminently calculated.

The advantages promised by it, are many. In the first place, it will tend to promote harmony and social intercourse, among its fellows. In the second, an interchange of sentiment on professional subjects, which cannot be otherwise than beneficial to all. In the third, its transactions, together with the communications that will, from time to time, be likely to be made to it, both from this and other countries, will contribute greatly to the literature of the art ; and, in the fourth and last place, the inducements which it proposes to hold out to genius, will have a tendency to awaken a new spirit of enquiry and physiological research, both into the theoretical and practical departments of the profession that may result in the increase of its usefulness — in short, to explode error, and establish correct doctrines and a uniform system of practice, are among the most prominent of its designs.

Of the projector of the Society, Dr. Hayden, its present President, it may not become us to speak. To the profession, however, having been a member of it for forty-three years, he is well known. As a man of science, and as an eminent practitioner, none stands higher, either in this or any other country. But however distinguished a reputation he may have gained as a dental practitioner, or as a man of science, the carrying into successful operation this project, will secure to him a fame that will out-live and out-shine even that. When he shall have been forgotten as a dental practitioner and physiologist, he will be remembered by his professional successors, as the *father of the* "*American Society of Dental Surgeons.*" Prominently connected with the Society, also, are the names of other distinguished men,—men, whose labours in this department of surgical knowledge, are too well, and too extensively known, to need any commendation from us. Those of Flagg, Parmly, Gardette, Harrington and Baker, as well as others which we could mention, have been long identified with the profession. Thus commenced, who can doubt that the efforts of the association will be crowned with success—the predictions of its enemies to the contrary notwithstanding.

Let it not be said, that, because it is opposed by a few, it will come to nought; but rather let those connected with it, put forth their energies to sustain and carry out its designs. Let them, by zealous and persevering exertions, bear down whatever of opposition may come in their way. Let them support and carry out the objects set forth in the instrument to which they have placed their signatures, and they will have peformed a work that shall signalize their names in all after times,—a work that will reflect upon themselves a credit of which they will never have cause to be ashamed, that will be recollected by those that shall come after them with pride, and that shall hereafter place the profession above reproach, and secure to its members the respect that is due to gentlemen and benefactors of mankind.

Having laid the foundation, let them go on with the superstructure, that, when they shall have passed from time, it may be in such a state of forwardness, that those who shall succeed them in the field of labour in which they have been, and are still engaged, may be induced to take hold and complete that which they shall have left unfinished.

The means for the bettering the condition of the profession, have been to us a subject of much reflection. To see its character elevated, has long been our most ardent desire, and through the agency and influence of this society, we trust that it will eventually be accomplished. We do not however, expect that it will effect an entire reformation in a single year, nor in five. To cleanse the profession from the leprosy of quackery will, we are persuaded, require considerable time, and can only be done as the public shall be furnished with the means of discriminating between those that are qualified to practice, and those that are not. But by the agency of this Society, it will gradually become possessed of these means. [BALT. ED.

